# Supplementary material for: Normalization of Muscle Strength Measurements in the Assessment of Cardiometabolic Risk Factors in Adolescents
Source: Int J Environ Res Public Health. 2021 Aug 10;18(16):8428. doi: 10.3390/ijerph18168428 (PMC8392172; doi:10.3390/ijerph18168428)
Supplement: Supplementary file 1 [file ijerph-18-08428-s001.zip › ijerph-1223408-supplementary.pdf]

International Journal of
Environmental Research and Public Health

Table S1. Statistical power for analysis from logistic regression for the relationship between adverse cardiometabolic conditions and muscle strength among males.

Outcomes	Absolute muscle strength (N)			Muscle strength normalized for body mass (N/kg)			Muscle strength normalized for BMI [N/(kg/m²)]			Muscle strength normalized for height (N/height)			Muscle strength normalized for fat mass (N/fat mass)		
	mean	SD	Power	mean	SD	Power	mean	SD	Power	mean	SD	Power	mean	SD	Power
**Dyslipidemia**															
No (n = 44)	682.2	25.6	0.99	10.6	0.3	0.47	31.4	1.1	0.99	40.4	1.5	0.99	80.3	5.5	0.30
Yes (n = 111)	733.1	13.4		10.7	0.3		32.5	0.7		42.8	0.7		79.0	3.7	
**Glucose imbalance**															
No (n = 139)	716.3	13.2	0.88	10.8	0.2	0.99	32.6	0.6	0.99	412.4	7.3	0.79	81.5	3.0	0.99
Yes (n = 16)	739.3	28.8		9.3	0.7		28.7	2.4		422.2	14.1		61.0	13.0	
**High blood pressure**															
No (n = 144)	718.4	12.9	0.07	10.8	0.18	0.99	32.5	0.6	0.99	413.1	7.1	0.08	80.7	3.2	0.99
Yes (n = 11)	722.5	31.8		9.4	0.7		28.4	2.2		415.5	17.6		62.3	11.0	
**Obesity**															
No (n = 119)	709.1	14.6	0.99	11.2	0.2	0.99	33.9	0.6	0.99	406.7	7.9	0.99	91.0	3.3	0.99
Yes (n = 36)	750.2	20.0		9.0	0.3		26.2	0.9		435.2	11.6		40.8	2.2	
**High inflammation**															
No (n = 142)	720.1	12.6	0.28	720.2	12.6	0.99	32.4	0.6	0.28	414.5	7.0	0.99	80.3	3.1	0.90
Yes (n = 13)	702.6	46.7		702.6	46.5		30.5	2.58		400.6	23.9		69.1	12.	

N: Newton—International System of Units; **SD**: Standard deviation.

Table S2. Statistical power for analysis from logistic regression for the relationship between adverse cardiometabolic conditions and muscle strength among females.

Outcomes	Absolute muscle strength (N)			Muscle strength normalized for body mass (N/kg)			Muscle strength normalized for BMI [N/(kg/m²)]			Muscle strength normalized for height (N/height)			Muscle strength normalized for fat mass (N/fat mass)		
	mean	SD	Power	mean	SD	Power	mean	SD	Power	mean	SD	Power	mean	SD	Power
**Dyslipidemia**															
No (n = 75)	445.4	14.2	0.99	8.1	0.2	0.99	20.1	0.6	0.99	277.5	8.9	0.95	37.1	1.7	0.99
Yes (n = 121)	453.6	8.2		7.5	0.1		19.4	0.4		281.5	4.7		31.2	1.4	
**Glucose imbalance**															
No (n = 159)	456.1	8.3	0.99	7.9	0.1	0.99	20.5	0.3	0.99	283.8	5.0	0.99	34.5	1.2	0.99
Yes (n = 37)	423.2	15.9		6.9	0.3		17.9	0.9		263.1	9.6		28.5	2.5	
**High blood pressure**															
No (n = 180)	445.0	17.2	0.99	7.7	0.1	0.42	19.8	0.3	0.95	277.0	4.3	0.99	33.6	1.1	0.19
Yes (n = 16)	52.1	40.2		8.0	0.7		21.6	2.0		312.8	24.8		32.4	4.0	
**Obesity**															
No (n = 131)	430.2	8.3	0.99	8.2	0.1	0.99	21.1	0.4	0.99	267.5	5.0	0.99	39.0	1.3	0.99
Yes (n = 65)	491	13.5		6.8	0.2		17.7	0.6		305.0	8.2		22.3	0.8	
**High inflammation**															
No (n = 154)	446.7	8.7	0.99	7.9	0.1	0.99	20.6	0.4	0.99	277.5	5.2	0.99	35.5	1.3	0.99
Yes (n = 42)	463.6	13.7		7.0	0.2		17.9	0.6		288.9	8.2		26.1	1.6	

N: Newton—International System of Units; **SD**: Standard deviation.

Table S3. Statistical power for analysis from multinomial logistic regression for the relationship between number of adverse cardiometabolic conditions and muscle strength according to sex.

Outcomes	Absolute muscle strength (N)			Muscle strength normalized for body mass (N/kg)			Muscle strength normalized for BMI [N/(kg/m²)]			Muscle strength normalized for height (N/height)			Muscle strength normalized for fat mass (N/fat mass)		
	mean	SD	Power	mean	SD	Power	mean	SD	Power	mean	SD	Power	mean	SD	Power
**Male**															
Number of cardiometabolic risk factors															
0 (n = 34)	686.0	30.2	0.99*	11.0	0.4	0.28*	32.6	1.3	0.99*	398.6	16.9	0.93*	87.6	6.4	0.13*
1 (n = 76)	715.3	17.6	0.99†	11.1	0.2	0.99†	33.8	0.7	0.99†	409.4	9.7	0.99†	88.6	3.9	0.99†
2 + (n = 45)	749.1	18.3	0.99‡	9.7	0.3	0.99‡	29.3	1.1	0.99‡	431.0	10.1	0.99‡	57.6	5.6	0.99‡
**Female**															
Number of cardiometabolic risk factors															
0 (n = 41)	427.8	16.5	0.94*	8.4	0.3	0.99*	21.6	0.7	0.99*	267.4	10.6	0.60*	40.9	2.1	0.99*
1 (n = 77)	437.9	11.1	0.99†	8.0	0.3	0.99†	20.7	0.5	0.99†	271.4	6.4	0.99†	37.0	1.9	0.99†
2 + (n = 78)	474.5	11.9	0.99‡	7.1	0.3	0.99‡	18.5	0.5	0.99‡	294.9	7.2	0.99‡	26.1	1.2	0.99‡

N: Newton—International System of Units; SD: Standard deviation; *: For the category 0 Vs. 1; †: For the category 0 Vs. 2; ‡: For the category 1 Vs. 2.

Table S4. Descriptive characteristics for the total sample and according to sex.

Variables	Total (n=351) mean±SD	Male (n = 155) mean±SD	Female (n = 196) mean±SD
**Height (cm)**	166.5±9.3	173.5±6.8*	160.6±6.2
**Body weight (kg)**	63.6±13.9	68.7±13.9*	59.6±12.4
**BMI (kg/m²)**	22.9±4.4	22.7±4.3	23.0±4.4
**TR (mm)**	14.7±6.0	10.9±4.2	17.8±5.4*
**SB (mm)**	12.8±5.8	10.6±4.1	14.5±6.4*
**∑TR+SE (mm)**	27.5±11.4	21.4±7.8	32.4±11.1*
**Body Fat (%)**	21.1 (7.4)	15.8 (5.6)	25.3 (5.8)*
**Fat mass (body weight x %BF/100)**	13.7±6.7	11.4±6.3	15.6±6.4*
**WC (cm)**	72.3±8.9	74.7±7.7*	70.4±9.3
**Systolic Blood Pressure (mm Hg)**	111.0±14.8	116.4±15.4*	106.7±12.7
**Diastolic Blood Pressure (mm Hg)**	68.2±9.6	67.4±9.8	68.8±9.4
**Cholesterol (mg/dL)**	148.3±32.9	136.9±30.5	157.5±31.8*
**HDL Cholesterol (mg/dL)**	47.3±11.3	43.6±8.7	50.3±12.2*
**LDL Cholesterol (mg/dL)**	89.4±27.9	82.9±26.8	94.7±27.7*
**Triglycerides (mg/dL)[a]**	58.0 [45.0 - 81.0]	53.0 [45.0 - 75.0]	61.0 [46.0 - 86.0]†
**Fasting glucose (mg/dL)**	80.3±7.1	81.7±7.3*	79.2±6.8
**Insulin (mU/L)[a]**	8.9 [6.9 – 12.4]	8.1 [6.0 – 10.8]	9.9 [7.3 – 13.1]†
**HOMA-IR (%)[a]**	1.8 [1.3 – 2.5]	1.7 [1.2 – 2.2]	1.9 [1.4 – 2.7]†
**C-reactive protein (mg/L)[a]**	1.2 [0.7 – 2.1]	1.1 [0.6 – 1.7]	1.4 [0.8 – 2.8]
**Absolute muscle strength (N)**	575.1±185.8	725.4±150.3*	455.3±107.9
**Muscle strength normalized for body mass (N/kg)**	9.1±2.5	10.7±2.2*	7.8±1.8
**Muscle strength normalized for BMI [N/(kg/m²)]**	25.6±8.6	32.5±7.2*	20.1±4.9
**Muscle strength normalized for height (N/height)**	342.5±99.1	416.8±83.0*	283.3±65.3
**Muscle strength normalized for fat mass (N/fat mass)**	53.9±25.5	79.0±29.3*	33.8±15.9
	% (95%CI)	% (95%CI)	% (95%CI)
**Ethnicity/Race**			
White	55.1 (42.5; 67.1)	48.2 (44.3; 52.2)	60.6 (41.7; 76.8)
Brown/Black/Yellow/Indigenous	44.9 (32.8; 57.5)	51.8 (47.8; 55.7)	39.4 (23.1; 58.3)
**Socioeconomic level**			
D-E	1.5 (0.7; 3.0)	1.9 (1.4; 2.6)	1.1 (0.2; 5.1)
C2	9.8 (3.0; 27.4)	7.0 (2.5; 6.9)	12.0 (9.2; 15.6)
C1	23.8 (17.9; 31.0)	23.3 (13.7; 36.8)	24.2 (21.8; 26.7)
B2	46.1 (41.5; 50.8)	47.8 (31.7; 64.4)	44.8 (39.5; 50.1)
B1	13.0 (8.2; 19.9)	15.3 (8.6; 25.6)	11.0 (2.2; 41.1)
A	5.8 (2.8; 11.9)	4.7 (2.8; 7.9)	6.9 (1.5; 25.2)
**Physical activity**			
Insufficiently active	87.6 (96.6; 88.5)	83.9 (81.8; 85.9)	90.5 (88.2; 92.4) ‡
Physically active	12.4 (11.5; 13.3)	16.1 (14.1; 18.2)	9.5 (7.5; 11.8)
**Muscle strength exercise**			
No	73.8 (66.9; 79.7)	63.3 (52.5; 72.9)	82.1 (76.0; 87.0) ‡
Yes	26.2 (20.3; 33.1)	36.7 (27.1; 47.5)	17.9 (13.0; 24.0)
**Balanced diet**			
Less frequent	87.9 (81.8; 92.2)	82.1 (68.4; 90.7)	92.6 (90.6; 94.1)‡
Frequent	12.1 (7.8; 18.1)	17.9 (9.3; 31.6)	7.4 (5.8; 9.4)

**Smoking**			
Yes	18.5 (13.0; 25.6)	16.6 (4.8; 44.1)	19.9 (16.6; 23.7)
No	81.5 (74.4; 87.0)	83.4 (55.9; 95.2)	80.1 (76.3; 83.4)
**Excess alcohol use**			
Yes	42.9 (31.0; 55.8)	42.6 (31.0; 55.1)	43.2 (30.6; 56.8)
No	57.0 (44.2; 69.0)	57.4 (44.7; 69.0)	56.8 (43.2; 69.4)
**Maturational Status**			
Prepubertal	11.0 (7.4; 16.1)	12.0 (8.0; 17.8)	10.2 (3.2; 28.0)
Pubescent	73.8 (56.3; 86.1)	73.9 (54.7; 86.9)	73.8 (56.1; 86.1)
Postpubertal	15.2 (6.9; 30.0)	14.1 (2.8; 47.5)	16.0 (10.2; 24.2)

N: Newton—International System of Units; SD: Standard deviation; CI: Confidence interval; BMI: Body Mass Index; TR: Triceps skinfold; SB: Subscapular skinfold; WC: Waist circumference; a: Median and Interquartile range; *: p value < 0.05 for Men vs. Women according to *t* test; †: p value < 0.05 for Men vs. Women according to Mann-Whitney test; ‡: p value < 0.05 for Men vs. Women according to Chi-squared test; .

Table S5. Number and frequency of individual and combined adverse cardiometabolic conditions for total sample and according to sex.

Variables	Total n (%)	Male n (%)	Female n (%)
**Obesity**			
No	250 (70.2)	119 (76.9)	131 (64.9)
Yes	101 (29.8)	36 (23.1)	65 (35.1)
**Dyslipidemia**			
No	119 (34.6)	44 (29.0)	75 (39.0)
Yes	232 (65.4)	111 (71.0)	121 (61.0)
**Glucose imbalance**			
No	298 (85.6)	139 (90.6)	159 (81.7)*
Yes	53 (14.4)	16 (9.4)	37 (18.3)
**High blood pressure**			
No	324 (92.9)	144 (93.0)	180 (92.8)
Yes	27 (7.1)	11 (7.0)	16 (7.2)
**High inflammation marker**			
No	296 (83.9)	142 (92.3)	154 (77.2)
Yes	55 (16.1)	13 (7.7)	42 (22.8)
**Number of cardiometabolic risk factors**			
**0**	75 (21.7)	34 (22.2)	41 (21.3)
**1**	153 (43.1)	76 (49.6)	77 (37.9)
**2**	73 (21.3)	29 (18.8)	44 (23.2)
**3**	32 (9.1)	11 (6.9)	21 (11.0)
**4**	17 (4.5)	05 (2.5)	12 (6.0)
**5**	01 (0.3)	00 (0.0)	01 (0.6)

*: *p* value < 0.05 for Male vs. Female according to Chi-squared test.

Table S6. Estimated predicted probability from the adjusted[a] multinomial logistic models for the relationship between number of adverse cardiometabolic conditions and muscle strength.

	Percentile	Predicted probability (95% CI)					
		**Male**			**Female**		
		0 cardiometabolic risk factor	1 cardiometabolic risk factor	2 + cardiometabolic risk factor	0 cardiometabolic risk factor	1 cardiometabolic risk factor	2 + cardiometabolic risk factor
**Absolute muscle strength (N)**	**10th**	0.28* (0.20; 0.35)	0.54* (0.40; 0.68)	0.18* (-0.04; 0.39)	0.26* (0.21; 0.32)	0.41* (0.32; 0.51)	0.32* (0.27; 0.37)
	**25th**	0.25* (0.18; 0.32)	0.53* (0.42; 0.64)	0.22*(0.04; 0.40)	0.25* (0.20; 0.29)	0.40* (0.28; 0.52)	0.35*(0.25; 0.44)
	**50th**	0.22* (0.16; 0.27)	0.51* (0.43; 0.58)	0.27* (0.15; 0.39)	0.23* (0.19; 0.27)	0.39* (0.23; 0.55)	0.38* (0.22; 0.54)
	**75th**	0.18* (0.15; 0.21)	0.47* (0.38; 0.56)	0.34*(0.25; 0.44)	0.19* (0.14; 0.24)	0.36* (0.13; 0.59)	0.44*(0.18; 0.71)
	**90th**	0.16* (0.14; 0.17)	0.44* (0.29; 0.59)	0.40* (0.25; 0.56)	0.17* (0.09; 0.24)	0.33* (0.04; 0.63)	0.50* (0.14; 0.86)

**Muscle strength normalized for body mass (N/kg)**	**10th**	0.13 (-0.01; 0.28)	0.40* (0.29; 0.51)	0.47* (0.32; 0.61)	0.13 (-0.02; 0.31)	0.29* (0.10; 0.47)	0.58* (0.21; 0.95)
	**25th**	0.17* (0.07; 0.28)	0.46* (0.37; 0.55)	0.37*(0.23; 0.50)	0.17* (0.02; 0.32)	0.34* (0.12; 0.55)	0.49*(0.13; 0.86)
	**50th**	0.23* (0.20; 0.25)	0.53* (0.44; 0.61)	0.25* (0.14; 0.35)	0.20* (0.10; 0.31)	0.38* (0.14; 0.61)	0.42* (0.08; 0.75)
	**75th**	0.26* (0.17; 0.36)	0.55* (0.43; 0.68)	0.18*(0.10; 0.26)	0.24* (0.19; 0.30)	0.41* (0.15; 0.68)	0.34*(0.05; 0.63)
	**90th**	0.29* (0.11; 0.47)	0.57* (0.39; 0.75)	0.14* (0.07; 0.21)	0.29* (0.18; 0.39)	0.44* (0.14; 0.75)	0.27* (0.03; 0.50)
**Muscle strength normalized for BMI [N/(kg/m²)]**	**10th**	0.16* (0.04; 0.27)	0.38* (0.30; 0.46)	0.46* (0.35; 0.57)	0.16* (0.03; 0.30)	0.29* (0.11; 0.48)	0.54* (0.23; 0.86)
	**25th**	0.19* (0.11; 0.27)	0.44* (0.37; 0.52)	0.37*(0.25; 0.48)	0.19* (0.08; 0.30)	0.33* (0.12; 0.54)	0.48*(0.16; 0.80)
	**50th**	0.22* (0.20; 0.25)	0.53* (0.43; 0.60)	0.26* (0.15; 0.36)	0.21* (0.13; 0.29)	0.37* (0.13; 0.60)	0.42* (0.11; 0.73)
	**75th**	0.25* (0.17; 0.34)	0.57* (0.43; 0.70)	0.18*(0.09; 0.27)	0.23* (0.19; 0.28)	0.41* (0.14; 0.68)	0.35*(0.07; 0.64)
	**90th**	0.27* (0.11; 0.43)	0.60* (0.41; 0.80)	0.13* (0.05; 0.20)	0.26* (0.19; 0.33)	0.45* (0.15; 0.75)	0.29* (0.04; 0.5)
**Muscle strength normalized for height (N/height)**	**10th**	0.27* (0.22; 0.31)	0.56* (0.38; 0.73)	0.17 (-0.04; 0.39)	0.26* (0.21; 0.31)	0.43* (0.36; 0.49)	0.31* (0.28; 0.34)
	**25th**	0.24* (0.19; 0.30)	0.53* (0.41; 0.66)	0.22*(0.04; 0.40)	0.24* (0.20; 0.29)	0.41* (0.31; 0.51)	0.34*(0.26; 0.42)
	**50th**	0.22* (0.16; 0.27)	0.50* (0.43; 0.57)	0.28* (0.16; 0.40)	0.23* (0.19; 0.26)	0.39* (0.24; 0.54)	0.38* (0.23; 0.53)
	**75th**	0.29* (0.15; 0.24)	0.47* (0.39; 0.54)	0.34*(0.25; 0.43)	0.20* (0.15; 0.25)	0.36* (0.13; 0.58)	0.44*(0.19; 0.70)
	**90th**	0.17* (0.14; 0.20)	0.43* (0.28; 0.57)	0.41* (0.26; 0.55)	0.18* (0.11; 0.24)	0.33* (0.04; 0.61)	0.49* (0.16; 0.83)
**Muscle strength normalized for fat mass (N/fat mass)**	**10th**	0.11 (-0.03; 0.25)	0.28* (0.18; 0.38)	0.61* (0.37; 0.84)	0.15 (-0.04; 0.34)	0.27 (-0.05; 0.60)	0.57* (0.06; 1.09)
	**25th**	0.15* (0.07; 0.24)	0.38* (0.30; 0.46)	0.47*(0.36; 0.57)	0.17* (0.02; 0.32)	0.31* (0.01; 0.61)	0.52*(0.07; 0.97)
	**50th**	0.23* (0.17; 0.28)	0.53* (0.36; 0.71)	0.24* (0.01; 0.47)	0.20* (0.11; 0.30)	0.36* (0.12; 0.61)	0.43* (0.10; 0.76)
	**75th**	0.28* (0.12; 0.45)	0.63* (0.56; 0.70)	0.09(-0.14; 0.31)	0.24* (0.19; 0.29)	0.42* (0.23; 0.61)	0.34*(0.15; 0.53)
	**90th**	0.31* (0.03; 0.59)	0.67* (0.48; 0.86)	0.02 (-0.07; 0.11)	0.26* (0.18; 0.33)	0.44* (0.27; 0.61)	0.30* (0.17; 0.44)

N: Newton – International System of Units; CI, confidence interval; * p value < 0.05 for the hypothesis that the corresponding marginal value is zero; a: Models adjusted for age, socioeconomic level, physical activity, muscle strength exercise, eating habits, smoking, excess alcohol use and maturational status.
